# Comparison of the Oral Health‐Related Quality of Life, Sleep Quality, and Oral Health Literacy in Sleep and Awake Bruxism: Results from Family Medicine Practice

**DOI:** 10.1155/2023/1186278

**Published:** 2023-09-30

**Authors:** Melike Mercan Başpınar, Çiğdem Mercan, Metin Mercan, Merve Arslan Aras

**Affiliations:** ^1^Department of Family Medicine, University of Health Sciences, Gaziosmanpaşa Training and Research Hospital, 34255 Gaziosmanpaşa, İstanbul, Turkey; ^2^Department of Oral and Maxillofacial Surgery, Istanbul University, Faculty of Dentistry, 34093, Fatih, Istanbul, Turkey; ^3^Department of Neurology, Bakirkoy Dr. Sadi Konuk Training and Research Hospital, Bakirkoy, 34147 Istanbul, Turkey; ^4^Department of Family Medicine, Ankara Etlik City Hospital, 06170 Yenimahalle, Ankara, Turkey

## Abstract

**Objectives:**

Bruxism is a common oral behaviour. This study aimed to compare oral health‐related quality of life, sleep quality, and oral health literacy in patients with and without possible sleep bruxism (SB) and awake bruxism (AB).

**Materials and Methods:**

A cross-sectional study including 249 volunteers was conducted in a family medicine clinic of a tertiary hospital in Istanbul, Turkey. The American Sleep Medicine Association Bruxism Diagnostic Criteria, Pittsburgh Sleep Quality Index (PSQI), Oral Health-Related Quality of Life (OHRQoL) tool, Decay Missing Filled Total Teeth (DMFT) score, and Health Literacy Dental Scale-Short Form (HeLD-14) were assessed by face-to-face interviews. Data were examined using Kruskal–Wallis and Mann–Whitney *U* tests, Spearman correlation, and logistic regression analysis.

**Results:**

The presence of SB and AB was detected as 41.4% and 21.7%, respectively, among 91 males and 158 females, with a mean age of 36.64 ± 11.60 years. Sleep and awake bruxers had a lower oral health-related quality of life (odds ratio (OR): 0.816, 95% confidence interval (CI) = 0.770–0.864 and OR: 0.923, 95% CI = 0.956–0.982, respectively). Poor sleep quality was detected 1.28 times higher in sleep bruxism (OR: 1.277, 95% CI = 1.152–1.415) and 1.14 times higher in awake bruxism (OR: 1.141, 95% CI = 1.230−1.058). The DMFT score was found to be 1.13 times higher in SB (OR: 1.129, 95% CI = 1.043–1.223). A higher HeLD-14 score was associated with a lower DMFT score (*p* < 0.001; *r* = −0.240). The oral health literacy score was lower in AB and SB groups than in patients without bruxism, but it was not statistically significant (*p* = 0.267, *p* = 0.376).

**Conclusion:**

A lower oral health-related quality of life and poor sleep quality would be expected in the presence of SB or AB. However, patients may not be aware of it unless asked by a physician regardless of oral health literacy level.

## 1. Introduction

Oral diseases and temporomandibular disorders lead to severe public health problems by their high prevalence and effects on the individual's quality of life [1]. Dental caries has been reported as a predictor of oral dental health-related quality of life (OHRQoL) [2]. However, this list may include bruxism as a predictor of both OHRQoL and sleep quality. The prevalence of bruxism is as high as 30% of the population [3]. Bruxism is an umbrella term to describe a multifaceted issue of masticatory muscle activities regarded as pathological when a person experiences possible negative consequences such as orofacial pain, damaged teeth, or temporomandibular joint interactions. So it could be a sign of a disorder in others [1, 3–5].

Recent studies have focused on the fact that bruxism may play a marked role in OHRQoL and sleep quality depending on oral dental health effects [4, 6]. While studies on sleep bruxism (SB) have often been discussed since the 1990s, few studies have been performed on the evaluation of awake bruxism (AB) [4, 7]. Awareness of bruxism in the general population is 15%–23% [8]. Improving the oral health literacy of patients may help in efforts to improve adherence to medical instructions, self-management skills, and overall treatment outcomes versus health inequality [9, 10]. However, no data exist on associations between bruxism and oral health literacy.

Dental professionals reported an increase in patients presenting with features of tooth wear, attributed to grinding and jaw clenching during the pandemic [11]. In this period, applications of patients with different kinds of complaints to family medicine centers also increased including oral dental health. So the aim of this study was primarily to determine the presence of possible SB or AB in family medicine practice and secondarily comparison of OHRQoL, sleep quality, DMFT (Decay + Missing + Filled + Teeth total) number, and oral health literacy level among patients with and without SB or AB.

## 2. Materials and Methods

This cross-sectional study was performed with 249 patients between June 2022 and July 2022 in a tertiary hospital family medicine outpatient clinic in Istanbul, Turkey. The scales to be applied to the participants were determined by consulting a neurologist for the diagnosis of bruxism and a dentist for oral and dental health. Bruxism occurs in two dimensions: sleep bruxism or awake bruxism. It is graded as “possible” based on patient reports, as “probable” based on clinical examination, and as “definite” by polysomnographic measurement [1]. According to the American Academy of Sleep Medicine, SB (sleep bruxism) and AB (awake bruxism) were diagnosed. The Pittsburgh Sleep Quality Index (PSQI) was used to assess sleep quality, the Oral Health-Related Quality of Life (OHRQoL) measure tool was used to determine the oral health-related quality of life, the Health Literacy Dental Scale-Short Form (HeLD-14) was used to measure the oral health literacy level, and the DMFT (Decay + Missed + Filled + Teeth total) number was used to dental control. After a family physician applied the study survey to patients chosen from the daily patient list order via https://randmizer.org, volunteers were invited to a dental control for DMFT evaluation during one day of the week. The frequency of going to the dentist and brushing teeth was asked to patients as an open-ended question. The answers were grouped by determining the headings in which the answers given were collected. The answers of the frequency of going to the dentist were collected under 2 headings. The first group responded “when there was a complaint,” and the second group was the group who went once or twice a year. Toothbrushing frequencies were collected under four headings: never, once daily, twice daily, and several times a week. Lastly, participants were asked which specialist they would refer to apply about teeth clenching or grinding. The physicians that patients would prefer to consult for their bruxism symptoms were collected as dentists, psychiatrists, neurologists, and family medicine specialists.

The study protocol was approved by the Ethics Committee of the Istanbul Gaziosmanpaşa Training and Research Hospital (08/06/2022-No: 89). All participants were informed about the study, and their written consent was obtained. This study has been performed in accordance with the ethical standards laid down in the 1964 Declaration of Helsinki and its later amendments.

### 2.1. Inclusion and Exclusion Criteria

Eighteen years and older volunteer adults were included in this study. Individuals diagnosed with bruxism who had a current oral dental treatment, dental prosthesis use, dental caries, psychiatric disability, neuromuscular disorders, diagnosed sleep disorder, malignancy, pregnancy, or any disease that could affect the nervous system or oral structure were excluded.

### 2.2. Sleep and Awake Bruxism Diagnosis

According to the last international consensus, the diagnosis of bruxism made by a self-report is classified as potential bruxism [5, 12]. The presence of complaints of clenching or grinding teeth during the daytime in the last six months was accepted as possible awake bruxism. According to the American Academy of Sleep Medicine, possible sleep bruxism (SB) was diagnosed if the participant reported or was aware of teeth-grinding sounds or teeth clenching during sleep or aware of abnormal tooth wear more than it should be. In addition, there must be one or more of the following symptoms upon awakening: temporary jaw muscle pain or tension in the morning, muscle weakness, fatigue at waking, hypertrophy of the masseter, hearing, or feeling a “click” in jaw joint upon awakening that disappears afterward.

### 2.3. Pittsburgh Sleep Quality Index (PSQI)

The validity and reliability of the PSQI Turkish language version consisting of 24 questions were made by Agargun et al. [13]. The sum of scores yields one global score of subjective sleep quality (range: 0–21). The sleep quality of a total score of <5 points is considered “good” and ≥5 points “poor.” Higher scores support worse sleep quality [14].

### 2.4. Oral Health-Related Quality of Life (OHRQoL) Scale

Oral health-related quality of life (OHQoL), which simply describes the influence of oral conditions on daily functioning, is an essential component of general health and well-being [15]. Turkish validation of the scale was carried out by Mumcu et al. [16]. The OHRQoL scale consists of 4 different categories and 16 questions with a structure of the Likert-type scale. It was thought that the negative effects of oral and dental diseases on the quality of life prevented individuals from noticing the positive effects of a healthy state in daily life. A low score on the OHRQoL scale indicates a low oral health-related quality of life. The lowest score that can be obtained from the scale is 16, and the highest score is 80 points [15, 16].

### 2.5. Health Literacy Dental Scale-Short Form (HeLD-14)

Oral health literacy was evaluated using the Health Literacy in Dentistry Scale-Short Form, originally developed and validated for the Indigenous Australian population. Item scores were recorded using a 5-point Likert scale, including response options ranging from “strongly disagree” to “strongly agree.” Higher scores show higher literacy [17, 18]. Turkish validation of the scale was corrected as 12 items [18, 19]. The Cronbach alpha coefficient of the scale was found to be 0.860. [18].

### 2.6. Statistical Analysis

Statistical software E-Picos Calculator was used to analyze the data. In the descriptive analysis, the number, percentage, mean, and standard deviation values were given. A *p* value of less than 0.05 was considered to be statistically significant. Mann–Whitney *U* and Kruskal–Wallis tests were used to compare nonnormally distributed continuous variable groups. Post hoc tests assessed the differences between multiple groups comparing. The Spearman test was used to assess correlations between scale scores. Binary logistic regression tests were used to explain age, PSQI, OHRQoL, and DMFT number effects related to the presence of SB and AB.

## 3. Results

The mean age of 249 patients was 36.64 ± 11.60 years, and 63.5% were female. The mean number of total decay, missed, and filled teeth was 7.35 ± 5.95. The sociodemographic characteristics of the participants and comparison of groups for PSQI, OHRQoL, DMFT, and HeLD-14 scores are given in Table 1. Participants who graduated from high school/university (59.0%) had higher DMFT and HeLD-14 scores than those who graduated from primary/secondary school (*p*=0.001, *p* < 0.001). In addition, low income versus moderate and high income was significant for a low HeLD-14 score (*p*=0.023, *p*=0.020). Although the frequency of the annual dentist visit number was not significant, the frequency of daily brushing habit was significant on OHRQoL, DMFT, and HeLD-14 scores. The distribution rate of daily brushing frequency was obtained as 4.4% nonbrushing, 16.4% brushing several times a week, 41.4% brushing once a day, and 37.8% brushing twice daily. Nondaily brushing habits versus once or twice-daily brushing habits aggravate a bad impact on OHRQoL, DMFT, and HeLD-14 scores (*p*=0.026, *p* < 0.001, *p* < 0.001, respectively). In addition, it was observed that those who brushed their teeth twice daily had the best scores in this study and a better oral dental health-related quality of life, oral health literacy, and less tooth loss than those who brushed their teeth once a day (*p*=0.016, *p* < 0.001, *p* < 0.001, respectively). The average PSQI score of participants was 7.35 ± 5.95 points, and it indicated that all had poor sleep quality as they had a score over 5 points.

Evaluation of sleep bruxism (SB) and awake bruxism (AB) group variables have been demonstrated in Table 2. The presence of SB and AB was obtained at 41.4% and 21.7%, respectively. The mean PSQI scores of SB and AB groups were 11.25 ± 5.02 and 11.70 ± 5.08 points, respectively. The presence of both SB and AB was significant for worse sleep quality and lower oral health-related quality of life (*p* < 0.001, *p* < 0.001). A higher DMFT score was significant for the presence of SB (*p* < 0.001). The oral health literacy score was lower in AB and SB groups than in patients without bruxism, but it was not statistically significant (*p*=0.267, *p*=0.376).

As shown in correlation test results in Table 3, there was no relationship between HeLD-14 and OHRQoL scores (*p*=0.073). A negative correlation between higher OHRQoL and poor sleep quality (*p* < 0.001; *r* = −0.350) and a negative correlation between a higher OHRQoL and lower DMFT score (*p*=0.001; *r* = −0.202) were reported. A higher HeLD-14 score was associated with a lower DMFT score (*p* < 0.001; *r* = −0.241).

In Table 4, binary logistic regression tests were used to explain independent factors affecting age, HeLD-14, PSQI, OHRQoL, and DMFT on the presence of sleep and awake bruxism. Variables were selected for the binary logistic model according to Table 2 results. The DMFT score was found to be 1.13 times higher among sleep bruxers (OR: 1.129, 95% CI = 1.043–1.223). Sleep and awake bruxers had a lower oral health-related quality of life (OR: 0.816, 95% CI = 0.770–0.864 and OR: 0.923, 95% CI = 0.956–0.982, respectively). Poor sleep quality was detected 1.28 times higher in sleep bruxism (OR: 1.277, 95% CI = 1.152–1.415) and 1.14 times higher in awake bruxism (OR: 1.141, 95% CI = 1.230−1.058).

**Figure 1:**
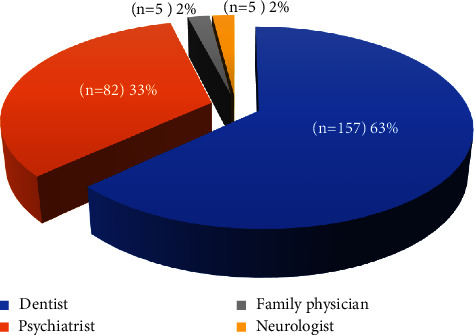
Distribution of health professional choices of patients for bruxism and symptoms.

Figure 1 shows the distribution of health professional choices of participants for bruxism and symptoms. It was indicated that participants with bruxism first thought of admitting a dentist (63.0%), a psychiatrist (33.0%), a neurologist (2.0%), or a family physician specialist (2.0%), respectively.

## 4. Discussion

In this present study including 249 patients of a family medicine clinic, the presence of SB and AB was 41.4% and 21.7%. SB and AB played negative roles in OHRQoL and sleep quality. SB was risk for the total decay, missing, filled teeth (DMFT) number. A reduced amount of the DMFT number was related to increased OHRQoL and oral health literacy level.

The presence of AB behaviours among healthy young adults has been reported within the range of 23–40% [4]. Câmara-Souza et al. found a 38.4% AB presence among college preparatory students [20]. Similarly, we found the presence of AB to be 21.7% as expected. In the study by Maluly et al., the prevalence of SB was 12.5% with questionnaires alone and 5.5% with confirmed SB by polysomnography. In addition, studies related to special groups showed that the bruxism rate might increase to 79% in the severe anxiety groups and 100% in the severe depression groups [6]. So then, the presence of SB and AB might have differed by a large range: 10%–13% for SB and 22%–31% for AB [21]; in younger populations, however, bruxism could be more frequent, affecting up to 40%–50% of studies' participants [3, 22]. Eksi Özsoy et al. found that 29% of all participants had self-reported AB and that 42.3% had self-reported SB [23]. In our study, we found that the presence of SB has a 41.4% rate.

AB is associated with psychological traits, whereas SB is a complex activity with multiple neurological interactions with other sleep-related conditions [4]. Tınastepe et al. reported that no difference in sleep quality was obtained among bruxers versus nonbruxers [24]. In our study, we found all participants had poor sleep quality, and bruxers had significantly worse sleep quality in both SB and AB groups' comparing results.

OHIP-14 (oral health impact profile), OIDP (oral impacts on daily performances), and GOHAI (geriatric oral assessment index) are the most widely validated instruments to evaluate OHRQoL [25]. In our study, the OHRQoL-UK scale was used, and the average score of OHRQoL among participants was moderate at 39.69 ± 13.79 out of 80 points. In the Norwegian adult sample, the frequency of dental visits, the number of teeth, age, and sex were related to OHRQoL, but dental health behaviour had no significant effect on OHRQoL in studies with the OHIP-14 tool [26]. Turcio et al. showed that bruxism (SB, AB, or both) had a positive correlation with lower oral health-related quality of life [27]. Tay et al. detected that 42.82% of the 2,417 participants reported either possible AB or possible SB and a significant association with poorer OHRQoL [28]. In our study, the OHRQoL score was best in the twice-daily brushing group and worse in sleep or awake bruxers than in nonbruxers. However, higher OHRQoL had a relationship with higher sleep quality and lower DMFT numbers. It was thought that SB might lead to poor sleep quality among bruxers by affecting OHRQoL depending on DMFT.

Previous studies have shown that oral health literacy is related to good OHRQoL maintained by demographic factors such as age, gender, education level, monthly income, nutrition, and daily living activities [29]. Our study tried to observe the effect of the oral health literacy level by the HeLD-14 score among bruxers versus nonbruxers. However, no significant difference was obtained.

Patients have said they preferred to apply mostly to a dentist (63%) or psychiatrist (33%) more than a family physician (2%) about bruxism signs. For the first time, our study searched a bruxism self-report during the routine visits of family medicine patients. Although a high percentage (41.4%) of the patients who come to the family physician have SB, a low percentage (2%) of the patients think to talk to the family doctor about bruxism complaints. Family physicians' awareness of bruxism is important because of many associations of bruxism with anxiety, depression, antidepressant-induced bruxism [30], headaches, earaches, and sleep disorders [31].

Our study possesses some limitations. First, the PSQI score average of the study group was in favor of generally poor sleep quality, so the PSQI results of bruxers might be over the scores in other study samples. We thought that the pandemic effect on sleep quality in the general population might lead to this result. Second, sleep quality and bruxism were evaluated with a subjective self-report of the patient. There are no doubts that PSG is a tool accepted by clinicians and researchers as the gold standard in the diagnosis of SB [32]. Another limitation was that the nicotine addiction of patients was not questioned. A meta-analysis demonstrated that nonsmokers have approximately 1.7 times better sleep quality than smokers [33]. So smoking may be a confusing factor in our study. However, the strength of this search is that logistic regression analysis was run to verify the impact of SB or AB on sleep quality, DMFT, and OHRQoL.

## 5. Conclusion

Within the limitations of the present study, it can be concluded that sleep bruxers have reduced OHRQoL, a higher amount of decay, missing, filled teeth, and poor sleep quality. However, patients may not inform bruxism symptoms unless family physician asks, regardless of the oral health literacy level. Moreover, there is a correlation between lower decay, missing, filled teeth and oral health literacy, but the subject demands in-depth research.

## Figures and Tables

**Table 1:** Sociodemographic characteristics of the participants and comparison of PSQI, OHRQoL-UK, DMFT, and HeLD-14 scores.

Variables	Groups	PSQI	OHRQoL	DMFT	HeLD-14
		Mean ± SD	Mean ± SD	Mean ± SD	Mean ± SD
Gender	Male (*n* = 91; 36.5%)	7.87 ± 4.39	41.55 ± 13.07	7.92 ± 6.01	35.69 ± 9.11
	Female (*n* = 158; 63.5%)	8.70 ± 4.83	38.62 ± 14.13	7.03 ± 5.91	38.81 ± 7.75

*p* value		0.229	0.095	0.138	**0.003**

Education	Primary/secondary school (*n* = 102; 41%)	8.01 ± 4.48	39.27 ± 12.47	9.10 ± 6.88	34.38 ± 9.73
	High school/university (*n* = 147; 59%)	8.66 ± 4.81	39.98 ± 14.69	6.14 ± 4.88	39.96 ± 6.44

*p* value		0.400	0.768	**0.001**	**<0.001**

Marital status	Single/divorced (*n* = 105; 42.2%)	8.37 ± 4.72	40.85 ± 15.14	6.12 ± 5.85	37.76 ± 8.63
	Married (*n* = 144; 57.8%)	8.41 ± 4.67	38.85 ± 12.72	8.25 ± 5.88	37.61 ± 8.25

*p* value		0.949	0.486	**<0.001**	0.777

Frequency of annual dentist visit	When having complaint (*n* = 199; 80%)	8.24 ± 4.69	39.83 ± 13.96	7.51 ± 5.75	37.49 ± 8.44
	Once/twice a year (*n* = 50; 20%)	9.00 ± 4.63	39.12 ± 13.26	6.74 ± 6.67	38.40 ± 8.26

*p* value		0.237	0.771	0.078	0.458

Frequency of daily brushing habit	^a^Never (*n* = 11; 4.4%)	10.64 ± 4.63	32.45 ± 8.44	14.27 ± 10.25	24.36 ± 12.02
	^b^Once daily (*n* = 103; 41.4%)	8.16 ± 4.51	37.61 ± 13.25	8.51 ± 6.13	36.88 ± 7.57
	^c^Twice daily (*n* = 94; 37.8%)	8.52 ± 4.84	42.47 ± 14.84	4.94 ± 4.16	41.15 ± 6.23
	^d^Several times a week (*n* = 41; 16.4%)	8.10 ± 4.73	40.49 ± 12.61	8.12 ± 5.13	35.27 ± 8.94

*p* value		0.355	**0.026**	**<0.001**	**<0.001**
			*P* ^a−b^=1.000	*P* ^a−b^=0.159	*P* ^a−b^=0.011
			*P* ^a−c^=0.022	*P* ^a−c^=0.003	*P* ^a−c^ < 0.001
			*P* ^a−d^=0.427	*P* ^a−d^=1.000	*P* ^a−d^=0.061
			*P* ^b−c^=0.016	*P* ^b−c^ < 0.001	*P* ^b−c^ < 0.001
			*P* ^b−d^=1.000	*P* ^b−d^=1.000	*P* ^b−d^=1.000
			*P* ^c−d^=1.000	*P* ^c−d^=0.002	*P* ^c−d^=0.001

Income level	^e^Low: income < expenses (*n* = 55; 22.1%)	8.76 ± 4.74	41.31 ± 16.73	9.53 ± 7.58	34.58 ± 9.75
	^f^Moderate: income = expenses (*n* = 151; 60.6%)	8.11 ± 4.37	39.40 ± 12.55	6.85 ± 5.40	38.32 ± 7.85
	^g^High: income > expenses (*n* = 43; 17.3%)	8.11 ± 4.37	38.65 ± 14.04	6.33 ± 4.78	39.37 ± 7.55

*p* value		0.647	0.841	0.059	**0.010**
					*P* ^e−f^=0.023
					*P* ^e−g^=0.020
					*P* ^f−g^=1.000

DMFT: Decay Missing Filled Total Teeth number; OHRQoL: Oral Health-Related Quality of Life measure; PSQI: Pittsburgh Sleep Quality Index; HeLD-14: Health Literacy Dental Scale-Short Form; SD: standard deviation. a: never toothbrushing; b: once-daily brushing; c: twice-daily brushing; d: several times a week toothbrushing; e: low-level income; f: moderate income; g: high-level income. ^*∗*^In each column, bold results indicate a statistically significant difference between groups (*p*  < 0.05).

**Table 2:** Evaluation of sleep bruxism (SB) and awake bruxism (AB) group variables.

Variables (mean ± SD)	Sleep bruxism		*p* value	Awake bruxism		*p* value
	(+) group (*n* = 103; 41.4%)	(−) group (*n* = 146; 58.6%)		(+) group (*n* = 54; 21.7%)	(−) group (*n* = 195; 78.3%)	
	Mean ± SD	Mean ± SD		Mean ± SD	Mean ± SD	
Age (year) (36.64 ± 11.60)	38.81 ± 11.30	35.11 ± 11.58	**0.013**	40.57 ± 9.68	38.58 ± 10.12	0.648
PSQI score (7.35 ± 5.95)	11.25 ± 5.02	6.38 ± 3.13	**<0.001**	11.70 ± 5.08	7.48 ± 4.13	**<0.001**
OHRQoL score (39.69 ± 13.79)	28.58 ± 7.82	47.52 ± 11.54	**<0.001**	29.22 ± 9.36	42.59 ± 13.44	**<0.001**
DMFT (7.35 ± 5.95)	8.91 ± 6.14	6.25 ± 5.58	**<0.001**	8.18 ± 6.42	7.12 ± 5.81	0.241
HeLD-14 score (37.67 ± 8.39)	37.07 ± 8.42	38.09 ± 8.38	0.267	36.56 ± 9.16	37.98 ± 8.17	0.376

DMFT: Decay Missing Filled Total Teeth number; OHRQoL: Oral Health-Related Quality of Life measure,, PSQI: Pittsburgh Sleep Quality Index; HeLD-14: Health Literacy Dental Scale-Short Form; SD: standard deviation. ^*∗*^In each column, bold results indicate statistically significant differences between groups (*p*  <  0.05).

**Table 3:** Evaluation of the correlations between age and HeLD-14, PSQI, OHRQoL, and DMFT scores.

Variables		Age	HeLD-14	PSQI	OHRQoL	DMFT
Age	*r*	—	−0.164	0.166	−0.178	0.371
	*p*	—	**0.009**	**0.009**	**0.005**	**<0.001**

HeLD-14	*r*	−0.164	—	−0.017	0.114	−0.241
	*p*	**0.009**	—	0.788	0.073	**<0.001**

PSQI	*r*	0.166	−0.017	—	−0.350	0.116
	*p*	**0.009**	0.788	—	**<0.001**	0.068

OHRQoL	*r*	−0.178	0.114	−0.350	—	−0.202
	*p*	**0.005**	0.073	**<0.001**	—	**0.001**

DMFT	*r*	0.371	−0.241	0.116	−0.202	—
	*p*	**<0.001**	**<0.001**	0.068	**0.001**	—

DMFT: Decay Missing Filled Total Teeth number; OHRQoL: Oral Health-Related Quality of Life measure, PSQI: Pittsburgh Sleep Quality Index; HeLD-14: Health Literacy Dental Scale-Short Form. ^*∗*^In each column, bold results indicate statistically significant differences between groups (*p*  <  0.05); *r*: Spearman correlation test rho value.

**Table 4:** Binary logistic regression test results explaining independent factors for the presence of sleep and awake bruxism.

Variables	Presence of sleep bruxism		Presence of awake bruxism	
	OR (95% CI)	*p* value	OR (95% CI)	*p* value
Age	0.989 (0.950–1.029)	0.580	0.986 (0.981–1.048)	0.413
DMFT	1.129 (1.043–1.223)	**0.003** ^*∗*^	1.007 (0.944–1.074)	0.840
OHRQoL	0.816 (0.770–0.864)	**<0.001** ^*∗*^	0.923 (0.956–0.982)	**<0.001** ^*∗*^
PSQI	1.277 (1.152–1.415)	**<0.001** ^*∗*^	1.141 (1.23–1.058)	**0.001** ^*∗*^

OR: odds ratio; CI: confidence interval; OHRQoL: Oral Health-Related Quality of Life measure; PSQI: Pittsburgh Sleep Quality Index. ^*∗*^*p* value significance level is <0.05.

## Data Availability

The data used to support the findings of this study are available from the corresponding author upon request.
